# Study on multi-scale oil displacement mechanism polymer/nanoparticle composite flooding

**DOI:** 10.3389/fchem.2025.1605416

**Published:** 2025-06-04

**Authors:** Bei Wei, Ningyu Zheng, Yu Xue, Jian Hou, Yongsheng Liu, Zhixin Guo, Xuwen Qin, Qingjun Du

**Affiliations:** ^1^ State Key Laboratory of Deep Oil and Gas, China University of Petroleum (East China), Qingdao, China; ^2^ School of Petroleum Engineering, China University of Petroleum (East China), Qingdao, Shandong, China

**Keywords:** polymer flooding, nano-SiO_2_, enhanced oil recovery, microscopic flooding experiments, core displacement experiment

## Abstract

Polymer flooding is a popular enhanced oil recovery (EOR) technique; however, conventional polymers face challenges such as large dosages and limited shear resistance. To address these issues, we proposed a polymer/nanoparticle composite flooding method and investigated its feasibility through laboratory experiments. We first characterized the rheological properties and interfacial tension of various polymer/nano-SiO_2_ composite systems and examined their microscopic morphology using scanning electron microscopy (SEM). Subsequently, we conducted two-dimensional microscopic flooding experiments to evaluate sweep efficiency and analyze residual oil distribution patterns. Finally, we performed core flooding experiments to compare injection pressure and recovery efficiency among different flooding systems. Results indicate that the presence of nano-SiO_2_ effectively enhanced the viscosity of the polymer system. The viscosity-increasing mechanism is nanoparticles adsorbing onto polymer molecular chains to form network structures. The polymer/nano-SiO_2_ composite system significantly increased sweep efficiency and promoted the transformation of residual oil from reticulated patterns to cluster, membrane, and punctiform patterns. Compared to polymer flooding, the polymer/nano-SiO_2_ composite system required a smaller amount of usage, effectively avoids environmental pollution, and showed better injectivity, achieving a 6% higher recovery while reducing injection pressure by up to 14%.

## 1 Introduction

Polymer flooding is a widely adopted enhanced oil recovery (EOR) technique, which involves the injection of polymer solutions into hydrocarbon reservoirs to improve recovery. The displacement fluid systems viscosity increases by adding polymers, which will help inhibit the viscous fingering and enhance the sweep coefficient (Mohsenatabar Firozjaii and Saghafi, 2020; Rellegadla et al., 2017; Abidin et al., 2012), and then improve oil recovery significantly. In addition, recent viscoelastic theories have demonstrated that polymer viscoelasticity can enhance microscopic displacement efficiency by dragging the remaining oil out in the blind end of pores (Sandiford, 1964; Wever et al., 2011; Jacobsen, 2017; Song and Hatzignatiou, 2022; Mirzaie Yegane et al., 2022; Mahajan et al., 2021; Raffa and Druetta, 2019). Ensuring high viscosity of polymer solutions is a prerequisite and guarantee for improving oil recovery in polymer flooding (Sheng, 2013). However, polymer structures are susceptible to mechanical, chemical, thermal, and biological degradation, resulting in viscosity reduction (Song et al., 2018). Mechanical degradation occurs at all stages of the injection-reservoir production process and is mainly influenced by shear stress (Gumpenberger et al., 2012; Puls et al., 2016; Zechner et al., 2013; Ghoniem et al., 1981; Seright, 1983). Reservoir conditions (temperature and salinity) also affect polymer performance by altering molecular chain conformations (Aliabadian et al., 2019). Previous studies have demonstrated that the mechanical degradation of polymers is enhanced under high-salinity conditions. (Karami et al., 2018; Chen et al., 2012). The high temperature induces multiple effects on the system, including the reduction of solvent viscosity, which consequently shortens the molecular relaxation time of polymers, the weakening of physical interactions such as hydrogen bonding and chain entanglements, and the contraction of isolated polymer chains (Hu et al., 1995; Modig et al., 2003). Biodegradation results from microbial enzymatic activity that degrade the polymeric chains, reducing their efficiency (Wu et al., 2024)^.^ Undoubtedly, ensuring the injectivity of polymers is another key factor in polymer flooding. Polymers are high-molecular materials with a certain radius of gyration. When injected into reservoirs with small pore throats, high-concentration polymers will generate additional flow resistance, resulting in excessively high injection pressure (Kang et al., 2022). Due to limitations in oilfield injection equipment, excessive pressure will result in the inability to inject polymers.

As mentioned above, to improve polymer flooding performance, there are two directions for improvement, i.e., improving the shear resistance, temperature resistance, and salinity resistance of the polymer, and reducing the injection pressure of reservoir. A method is polymer/nanoparticle composite flooding, in which the polymer is a hydrophobically associative water-soluble polymer (HAWP) that has good temperature resistance and salinity resistance, and the nanoparticles can adsorb onto the rock surface and decrease the injection pressure. In the following, we will focus on the literature related to nanoparticles and look for whether there are some other interactions between polymers and nanoparticles in such a composite system.

Nanoparticles possess unique properties, including high specific surface area, unique chemical reactivity, and active surfaces. These characteristics enable them to adsorb onto reservoir surfaces, alter wettability, and reduce flow resistance of injection water, thereby enhancing waterflooding efficiency (Tafur et al., 2022). Currently, the most widely used nanoparticles in petroleum exploitation include metal oxide and inorganic nanoparticles, such as Al_2_O_3_, MgO, ZrO_2_, and Fe_2_O_3_ (Sun et al., 2023). Although they have excellent mechanical properties, temperature tolerance and salt resistance, their high cost and retention in the formation are still significant limiting factors. In contrast, SiO_2_ nanoparticles have attracted much attention due to their excellent performance and good chemical compatibility with sandstone formations (Alnarabiji and Husein, 2020).The nanoscale dimensions of these particles result in enhanced surface activity and specific surface area, leading to significant reductions in interfacial tension (IFT) (Eker et al., 2024; Cutroneo et al., 2021; Alvarez et al., 2013; Yekeen et al., 2020). Studies have confirmed that nanoparticles can alter or reverse rock surface wettability, increase oil contact angles, enhance oil displacement efficiency, and stabilize oil-water emulsions through interfacial adsorption (Bergfreund et al., 2019; Sharma et al., 2015). Further studies have revealed that under injection pressure, nanoparticles self-assemble into ordered microscopic layered structures within pore spaces, generating wedge-film structures at rock surface-oil droplet interfaces. The formation of this wedge-film structure increases the separation force, making it easier for the oil droplets to be detached (Trokhymchuk et al., 2001). Nanoparticles can effectively mitigate the challenges associated with high injection pressure and poor injectability in low-permeability reservoirs. (Zhao et al., 2024; Dai et al., 2015). Recent investigations have demonstrated synergistic effects between nanoparticles and chemical flooding in enhancing oil recovery. Adding nanoparticles into polymer flooding systems increased injection fluid viscosity, improved mobility ratio, and enhanced sweep efficiency (Cheraghian, 2015; Cheraghian and Hendraningrat, 2016; Rueda et al., 2020). Nanoparticle-polymer solutions demonstrate superior resistance to temperature and salinity effects compared to conventional polymer solutions (Elhaei et al., 2021; Corredor et al., 2019). Gbadamosi et al. (2019) found that the use of a combined system of HPAM and Al_2_O_3_ nanoparticles can further increase oil recovery by an additional 11.3% compared to using polyacrylamide (PAM). Overall, the nanoparticle-assisted polymer flooding technique exhibits superior performance compared to polymer flooding.

While extensive research has focused on nanoparticle modification, characterization, nanofluids stability, and macroscopic displacement efficiency, limited attention has been paid to microscopic residual oil mobilization in polymer/nanoparticle composite flooding systems. In this study, we optimized the formulation and characterized the performance of a hydrophobically associative water-soluble polymer (HAWP)/SiO_2_-nanoparticles composite system. Displacement experiments at the microscopic and core scales were carried out to study the microscopic oil displacement mechanisms of the composite system and improve the efficiency of oil recovery.

## 2 Material and methods

### 2.1 Materials

#### 2.1.1 Simulated formation water and crude oil

We prepared simulated formation water based on the reservoir conditions of Shengli Oilfield. The mineralization degree reached 19,334 mg/L, with calcium and magnesium ions constituting 514 mg/L. Composition of chemical agents in simulated formation water: 17.786 g/L NaCl, 1.14 g/L CaCl_2_, 0.86 g/L MgCl_2_·6H_2_O. We obtained all analytical grade chemical reagents from Sinopharm Chemical Reagent Co., Ltd. (Shanghai, China). To prepare the experimental crude oil, we diluted dehydrated crude oil with kerosene until its viscosity reached 42 mPa·s at ambient temperature (25°C).

#### 2.1.2 Polymer and nano-SiO_2_

Shandong Noel Biotechnology Co., Ltd. (Shandong, China) provided the hydrophobically associating water-soluble polymer (HAWP) for this study. To achieve stable dispersion of SiO_2_-nanoparticles in the composite system, SiO_2_-nanoparticles modified by surface silanization were selected (later referred to as SiO_2_), with particle sizes ranging from 20 to 30 nm.

### 2.2 Experimental methods

#### 2.2.1 Compounding of polymer/nano-SiO_2_ particle composite fluid

To obtain the polymer stock solution, we dissolved HAWP powder in the simulated formation water to achieve a mass concentration of 5,000 ppm and allowed the solution to age for 24 h. By diluting the stock solution, we prepared HAWP solutions at 1,000 ppm, 1,500 ppm, and 2,000 ppm. The addition of varying amounts of SiO_2_ to these solutions got composite systems containing 0.05 wt%, 0.1 wt%, 0.3 wt%, 0.5 wt%, and 1.0 wt% SiO_2_ at each HAWP concentration.

#### 2.2.2 Surface microstructure morphology observation

To observe microscopic morphology more clearly, we prepared two additional composite systems by diluting 5,000 ppm HAWP solution to 3,000 ppm(0.3 wt%) with simulated formation water and adding SiO_2_ to achieve concentrations of 0.5 wt% and 1.0 wt%, that is, the ratios of HAWP to SiO_2_ were 3:10 (0.3 wt%:1 wt%) and 3:5 (0.3 wt%:0.5 wt%) respectively. Then we examined the surface microstructure of the HAWP solution, SiO_2_, and the composite systems using scanning electron microscopy.

#### 2.2.3 Interfacial tension measurement

We selected four composite systems to test the interfacial tension: 2,000 ppm HAWP with SiO_2_ concentrations of 0.05 wt%, 0.1 wt%, 0.3 wt%, and 0.5 wt%. Before measurement, the composite systems underwent ultrasonic dispersion. Then we measured their interfacial tension with oil using an interfacial tensiometer at reservoir temperature (75°C) with a rotation speed of 6,000 r·min^−1^.

#### 2.2.4 Rheological testing

We measured the viscosity, temperature-viscosity relationships, and rheological behavior of different systems using an Anton Paar M302 high-temperature and high-pressure rheometer. To accurately simulate reservoir conditions, the following tests were conducted: The influence of different formulations on composite system viscosity was tested at reservoir temperature (75°C) and wellbore shear rate (7.34 s^−1^); The effect of temperature on composite system viscosity was investigated from 25°C–90°C at wellbore shear rate (7.34 s^−1^); The impact of shear rate on composite system viscosity was examined at shear rates of 3.5–100 s^-1^ at reservoir temperature (75°C).

#### 2.2.5 Investigation of microscopic oil displacement mechanism

**FIGURE 1:**
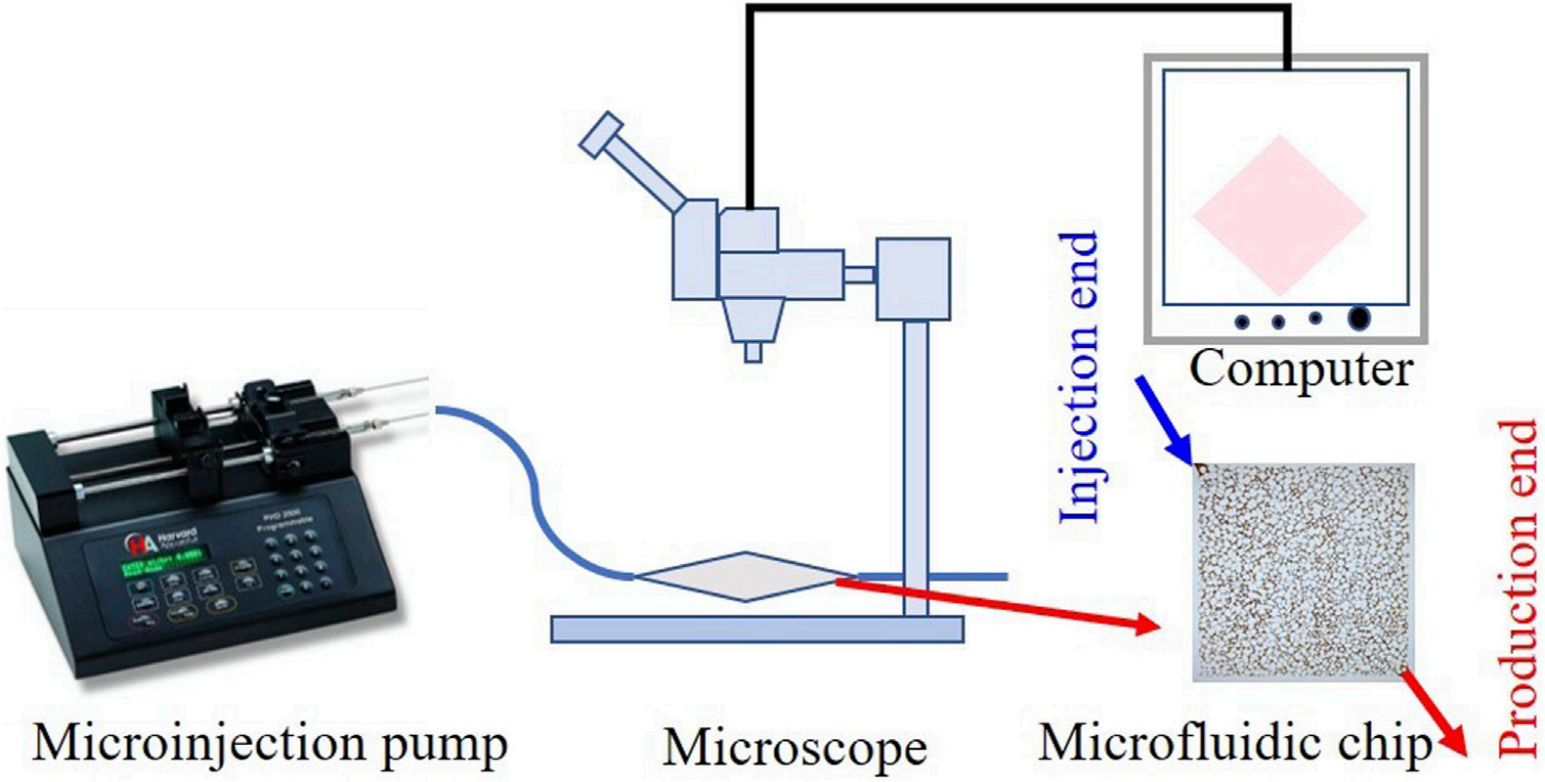
Microfluidic experimental device.

The microscopic oil displacement experiments utilized a microfluidic device comprising a microinjection pump, microfluidic chip, microscope, and computer (Figure 1). The microfluidic chip (45 mm × 45 mm) with pore diameters ranging from 20–80 μm. We investigated four displacement systems: formation water, 0.1 wt% SiO_2_, 2,000 ppm HAWP, and 2,000 ppm HAWP +0.1 wt% SiO_2_, each stained with eosin.

The experimental oil was injected into the microfluidic chip at an injection rate of 0.5 mL/min until saturation was achieved. Subsequently, a displacement agent was injected at a rate of 0.5 mL/min to perform displacement until the water cut reached 98%, at which point injection was stopped. The process was repeated four times using different oil displacement systems to observe the displacement effects.

#### 2.2.6 Core flooding experiments

**FIGURE 2:**
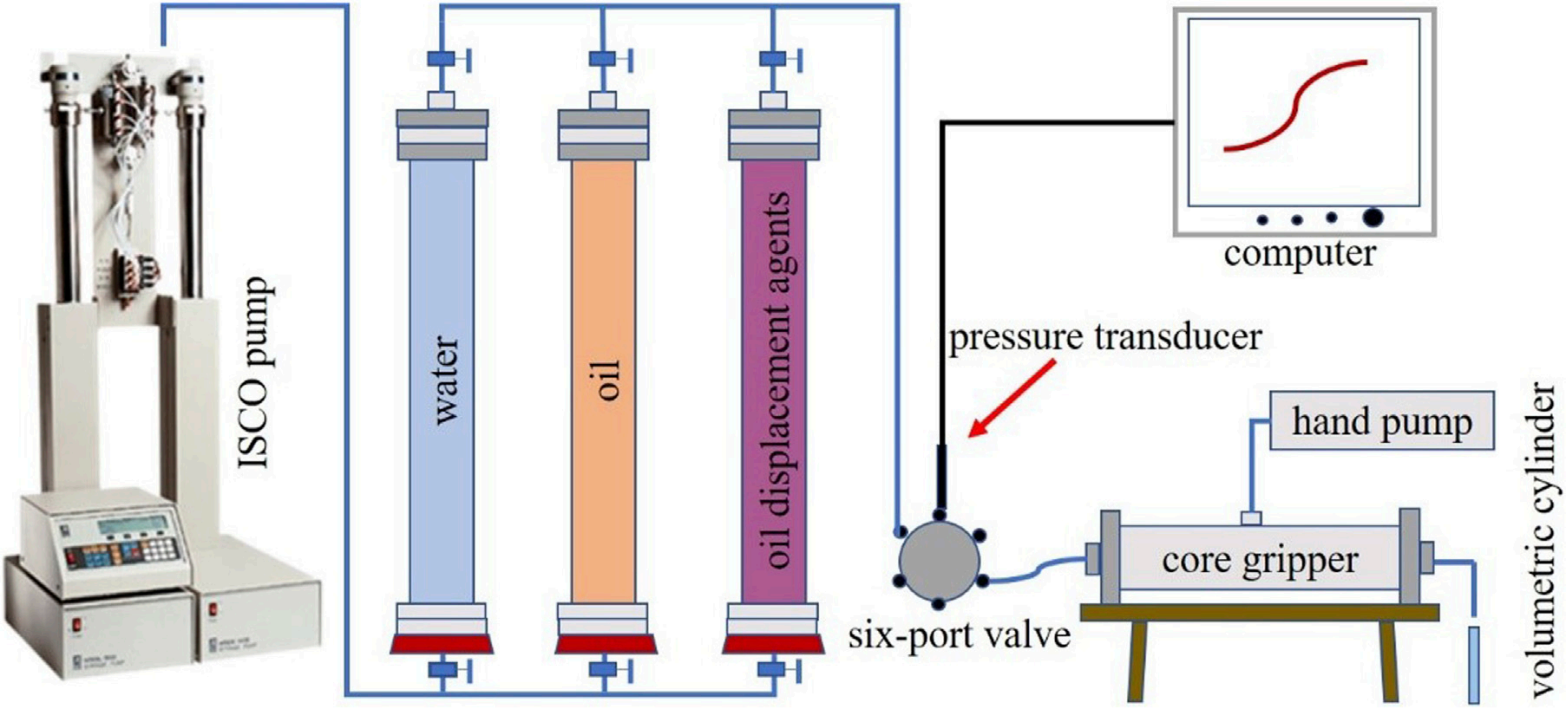
Core flooding experimental apparatus.

Core flooding experiments employed the same displacement systems as the microscopic studies. The experimental setup (Figure 2) incorporated an ISCO pump (Teledyne ISCO, United States), three containers, a core holder, a six-way valve, a pressure transducer, and a hand pump.

This system is mainly targeted at medium-high permeability and high water-cut oil reservoirs. We conducted experiments using standard sandstone cores (permeability: 400 mD, diameter: 25 mm, length: 100 mm). After the core was saturated with crude oil, water flooding was carried out. Water flooding was carried out until the water content reached 98%, and the corresponding chemical flooding was carried out until the water content reached 98% for subsequent water flooding. Following the completion of each experimental run, the oil displacement systems were replaced, and the aforementioned procedures were repeated. Throughout the flooding process, injection pressure variations were continuously monitored, and effluent samples were collected every 3 min for oil recovery and water content analysis.

## 3 Results and discussion

### 3.1 Characterization of basic properties of the composite system

#### 3.1.1 Microscopic morphology analysis

**FIGURE 3:**
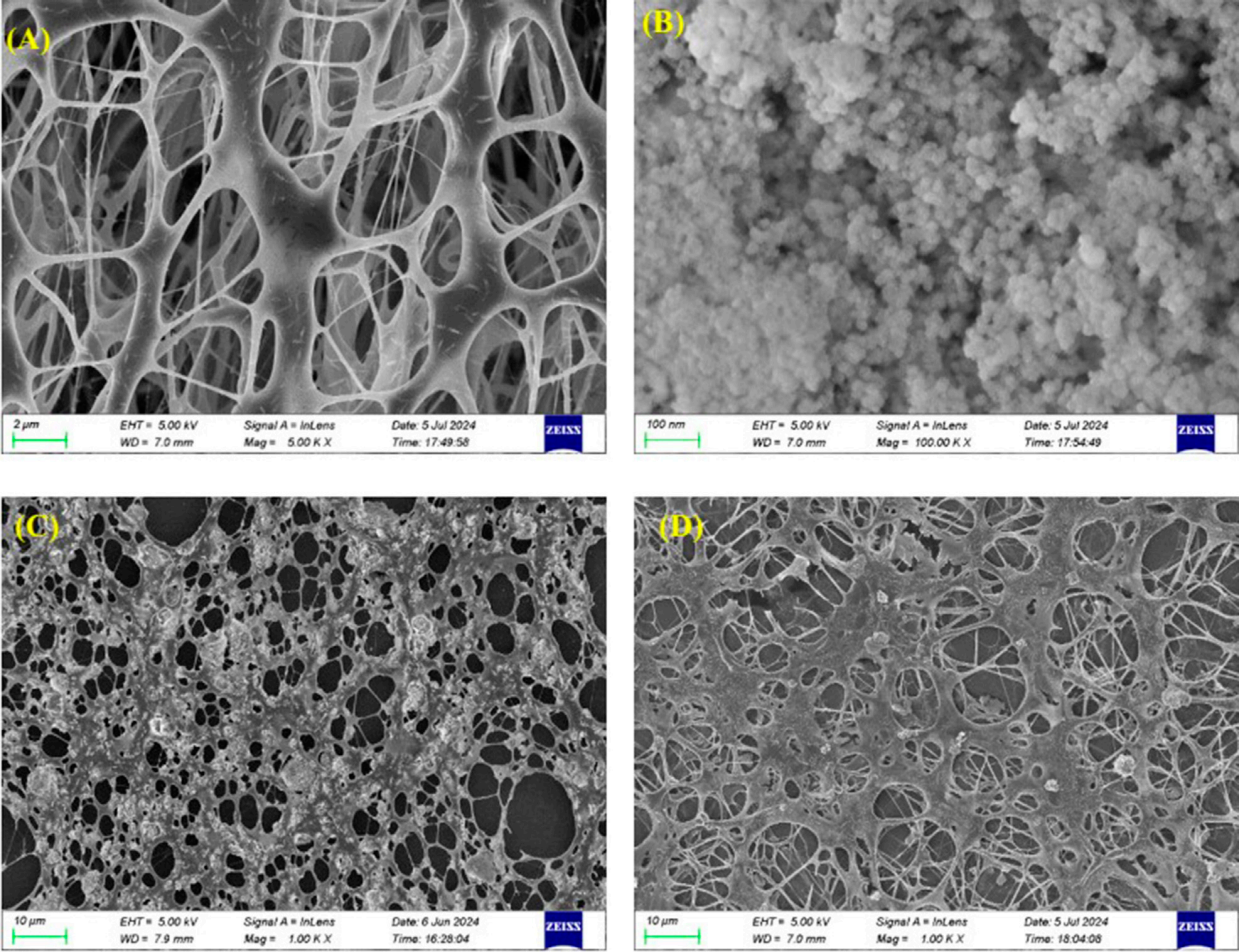
SEM micrographs of **(A)** 3000 ppm HAWP, **(B)** SiO_2_, **(C)** 3000 ppm HAWP +1.0 wt% SiO_2_ composite system, and **(D)** 3000 ppm HAWP +0.5 wt% SiO_2_ composite system.

Figure 3 shows scanning electron microscopy (SEM) images of (a) HAWP, (b) SiO_2_, (c) 3,000 ppm HAWP +1.0 wt% SiO_2_ (3:10), and (d) 3,000 ppm HAWP +0.5 wt% SiO_2_ (3:5) systems.

SEM image observations revealed the microstructural morphology of the polymer solutions and the SiO_2_ surface. The polymer molecules formed network structures. The SiO_2_ particles showed a spherical shape with diameters ranging from 20–30 nm and maintained a specific surface area of ≥120 m^2^/g.

Furthermore, SiO_2_ combined with HAWP to form stable composite systems, which increased the system viscosity through adsorption and bridging effects. As shown in Figure 3C, When the ratio of HAWP to SiO_2_ is 3:10, SiO_2_ aggregates were distributed throughout the polymer network structure, showing aggregate sizes between 2–3 μm, with a few aggregates reaching 4–5 μm. In comparison, when the ratio of HAWP to SiO_2_ is 3:5, SiO_2_ particles formed smaller aggregates of approximately 1–2 μm and displayed a more uniform distribution within the polymer network structure, indicating better compatibility between the nanoparticles and polymer.

#### 3.1.2 Interfacial tension analysis

**FIGURE 4:**
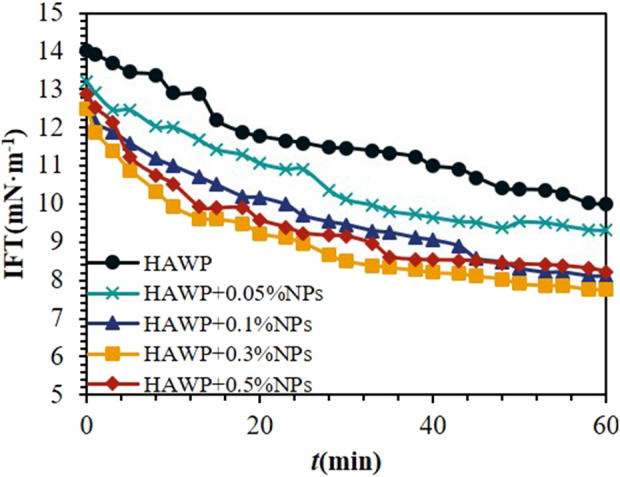
Dynamic interfacial tension profiles of 2000 ppm HAWP solutions with varying nano-SiO_2_ concentrations.

Figure 4 shows the dynamic interfacial tension curves of HAWP/SiO_2_ composite systems at different SiO_2_ concentrations. The addition of nano-SiO_2_ reduced the interfacial tension between the HAWP solution and crude oil. The polymer chains adsorbed onto the nano-SiO_2_ surface through hydrogen bonding. The nano-SiO_2_ particles became encapsulated within the free polymer chains. Both components adsorbed at the oil-water interface, leading to lower interfacial tension.

The oil-water interfacial tension continuously decreases as SiO_2_ mass concentration increases, exhibiting the most significant reduction at lower SiO_2_ concentrations. The interfacial tension drops to its minimum value at a SiO_2_ concentration of 0.3 wt%. The system maintains good stability when the SiO_2_ mass fraction remains below 0.3 wt%, and increasing the SiO_2_ mass fraction enhances its interfacial tension reduction capability. However, when the SiO_2_ mass fraction exceeds 0.3 wt%, the system stability declines, causing partial aggregation and failure of SiO_2_ particles, which weakens their ability to reduce interfacial tension. However, compared with surfactants, the magnitude of its interfacial tension reduction is relatively small, only decreasing by 2–3 mN m^−1^. While surfactants can reduce the interfacial tension to 10^–2^ – 10^–3^ mN m^−1^ (Negin et al., 2017). Therefore, reducing the interfacial tension is not the main mechanism for the composite system to improve the oil recovery rate. Considering factors such as cost comprehensively, a lower SiO_2_ concentration should be chosen.

#### 3.1.3 Rheological analysis

**FIGURE 5:**
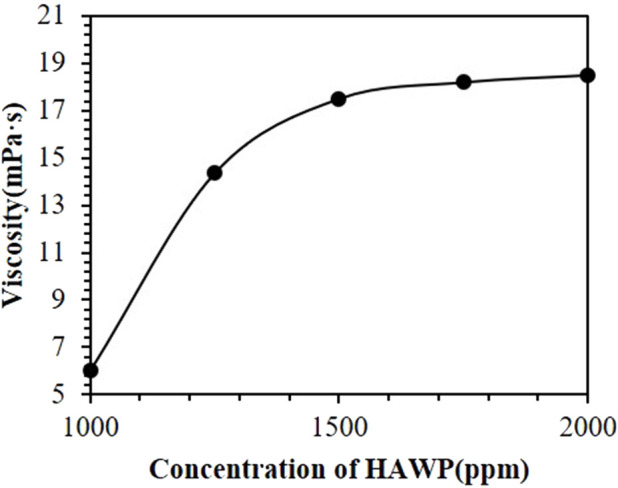
Viscosity of HAWP solutions as a function of concentration at 75°C.

Figure 5 shows the viscosity of HAWP at 75°C under a shear rate of 7.43 s^−1^. The viscosity increased with higher polymer concentration. This increase occurred because polymer molecules formed network structures. At higher concentrations, polymer chains became more entangled, resulting in more stable network structures. Therefore, polymer viscosity increased with concentration at the same shear rate.

When the HAWP concentration increased from 1,000 ppm to 1,500 ppm, the viscosity increased by 191.7%; however, as the concentration increased from 1,500 ppm to 2,000 ppm, the viscosity increment was only 5.7%. These results suggest that the polymer viscosity is more sensitive to concentration changes at lower concentrations, whereas the viscosity increment becomes less significant above a certain concentration threshold. This is mainly because the high-valence ions (such as Ca^2+^ and Mg^2+^) in high salinity water compress the electric double layer through the charge screening effect, causing the molecular chains to curl up prematurely. The hydrophobic groups preferentially undergo intramolecular association (rather than intermolecular association). At low concentrations, the molecular chains form a dynamic intramolecular association-dissociation cycle through instantaneous contact, and a slight change in concentration can significantly affect the viscosity. High salinity enhances the polarity of the solution and weakens the ability of the molecular chains to stretch, resulting in a significant increase in the critical association concentration. Even if the concentration increases, the intermolecular association network is difficult to form due to salt inhibition, and the viscosity cannot show the conventional exponential growth.

**FIGURE 6:**
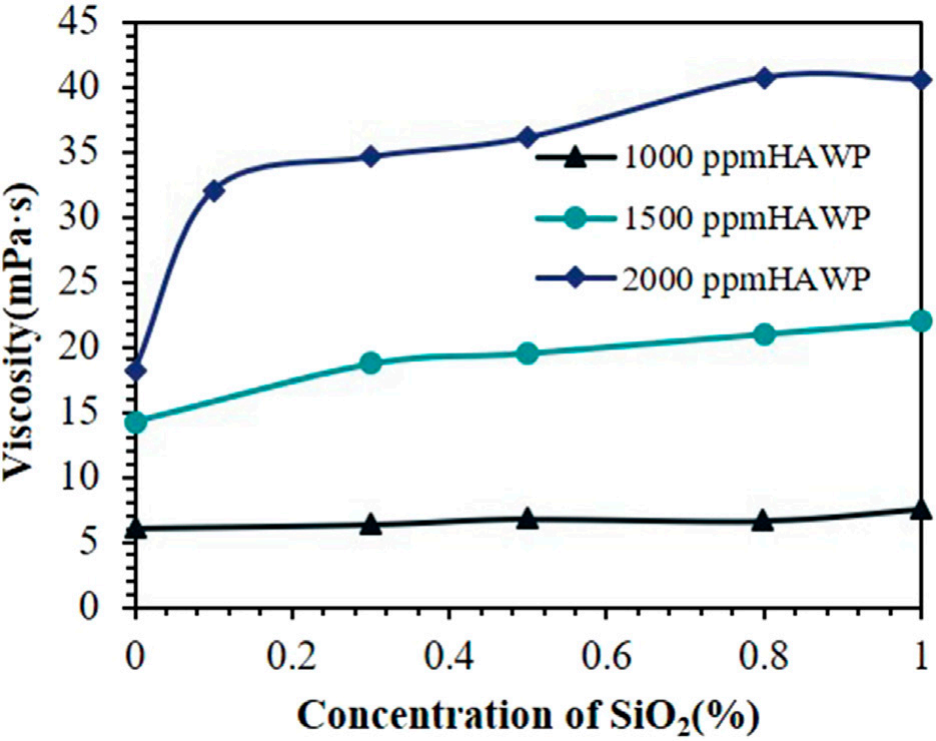
Effect of SiO_2_ concentration on the viscosity of composite systems with different HAWP concentrations.

Figure 6 shows the viscosity variations of composite systems at 75°C, where we added different mass fractions of SiO_2_ into HAWP solutions at various concentrations. The results demonstrate that the viscosity of the composite systems increases with rising SiO_2_ content. This increasing trend is more pronounced at higher polymer concentrations. The figure shows that in the 2,000 ppm HAWP + SiO_2_ composite system, the maximum slope occurs at 0.1 wt% SiO_2_ concentration. This indicates that SiO_2_ nanoparticles at 0.1 wt% demonstrate the most significant tackifying effect on 2,000 ppm HAWP. In practical applications, considering that nanoparticles may still be adsorbed in the formation, a higher concentration can be selected.

**FIGURE 7:**
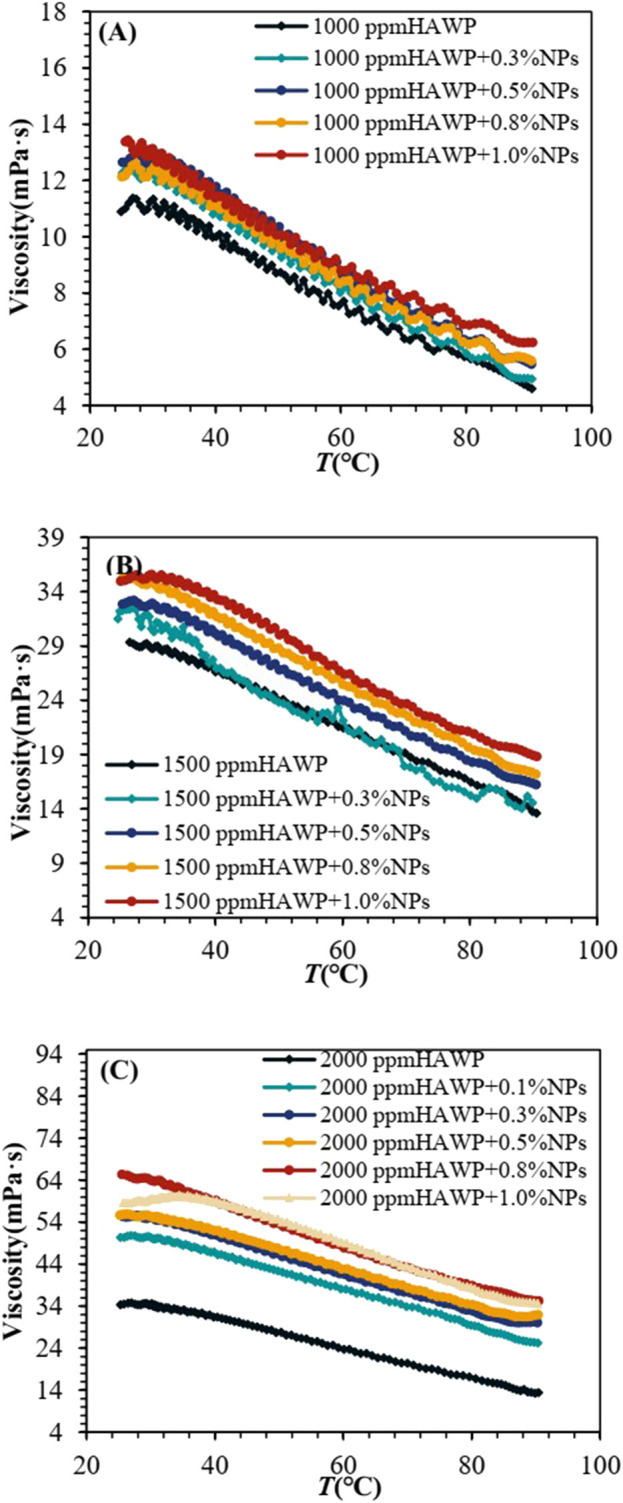
Temperature-viscosity curves of SiO_2_-HAWP composite systems at varying HAWP concentrations: **(A)** 1000 ppm HAWP, **(B)** 1500 ppm HAWP, and **(C)** 2000 ppm HAWP.

Figure 7 shows the viscosity variations of composite systems with different HAWP-to-SiO_2_ ratios as a function of temperature (25°C–90°C) at a constant wellbore shear rate of 7.34 s^−1^. The experimental results demonstrated that the viscosity of all composite systems exhibits a consistent declining trend with increasing temperature.

However, the viscosity of the HAWP/SiO_2_ composite system remained higher than that of HAWP. Furthermore, the viscosity increased with increasing SiO_2_ concentration. This phenomenon occurred because SiO_2_ could form a stable composite system with HAWP, enhancing the polymer chain network structure through adsorption and bridging effects, thereby increasing its viscosity. As shown in Figure 7C, when the HAWP concentration was 2,000 ppm, the temperature-viscosity curves of systems containing 0.8wt% and 1.0wt% SiO_2_ nearly overlapped. This indicated that beyond a certain threshold of SiO_2_ concentration, the viscosity-enhancing effect became insignificant, accompanied by decreased system stability, and SiO_2_ nanoparticles aggregated and lost their effectiveness.

**FIGURE 8:**
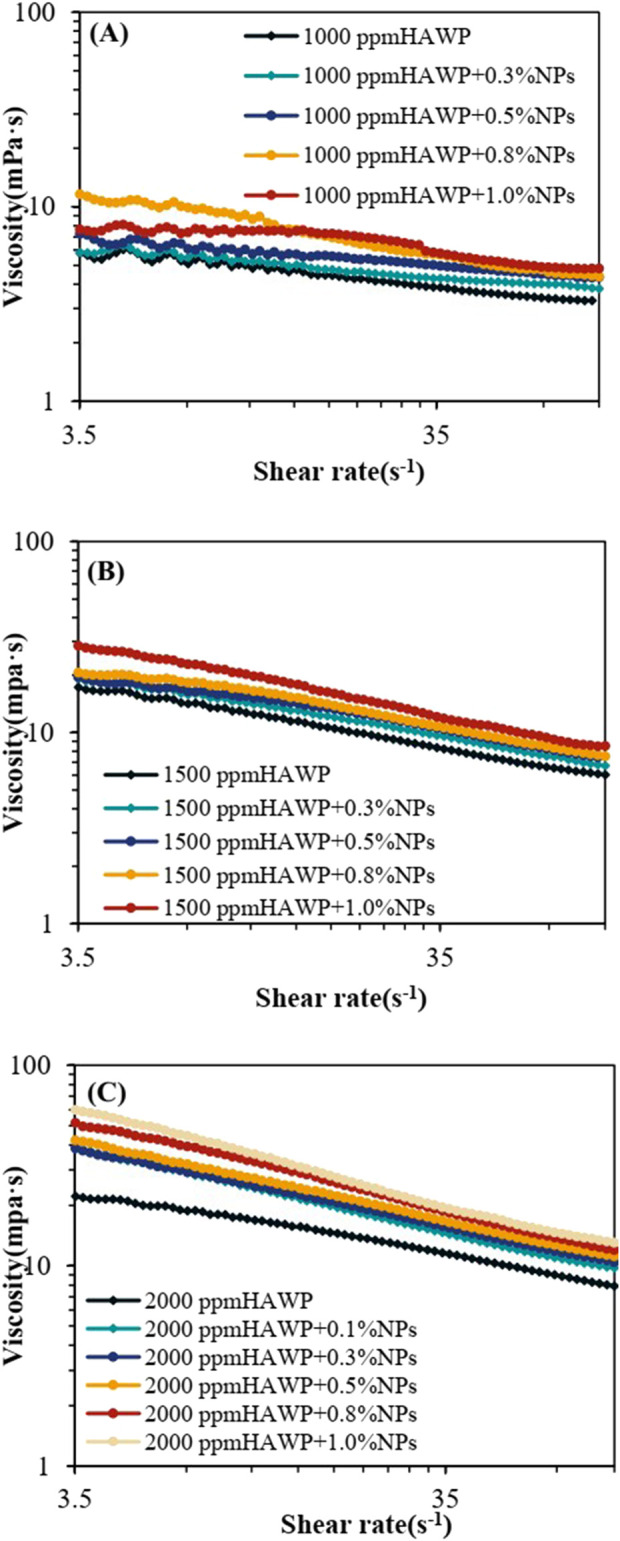
Shear rate-viscosity profiles of SiO_2_-HAWP composite systems at varying HAWP concentrations: **(A)** 1000 ppm HAWP, **(B)** 1500 ppm HAWP, and **(C)** 2000 ppm HAWP.

Figure 8 shows the viscosity variations of composite systems with different ratios as a function of shear rate (3.5–100 s^−1^) at reservoir temperature (75°C). The experimental results demonstrated that all composite systems exhibited a decreasing trend in viscosity with increasing shear rate. Systems containing SiO_2_ show higher viscosity compared to HAWP solutions of the same concentration, and the viscosity increased with increasing SiO_2_ concentration.

However, the thickening effect of SiO_2_ varies across different HAWP concentrations. As shown in Figures 8A, B, the viscosity-shear rate curves of composite systems with 1,000 ppm and 1,500 ppm HAWP demonstrate uniform distribution with increasing SiO_2_ mass concentrations. Figure 8C reveals that in the 2,000 ppm HAWP composite system, the addition of 0.1 wt% SiO_2_ leads to a rapid increase in the viscosity-shear rate curve. Further increases in SiO_2_ concentration result in the rising speed of the viscosity slows down. This indicates that SiO_2_ at 0.1 wt% concentration exhibits the most pronounced tackifying effect on 2,000 ppm HAWP.

While the addition of SiO_2_ did not fully address the viscosity reduction of HAWP solutions at high temperatures and shear rates, it significantly enhanced the overall viscosity of the composite systems. The viscosity increased with rising SiO_2_ concentrations, with 0.1 wt% SiO_2_ showing the most significant thickening effect on 2,000 ppm HAWP. Considering cost-effectiveness and performance, the 0.1 wt% SiO_2_ + 2,000 ppm HAWP composite system was selected as the optimal flooding system.The viscosity of this system at 75°C under a shear rate of 7.43 s^−1^ is 1.73 times that of the polymer with the same concentration under the same conditions, which can effectively reduce the usage amount of the polymer.

### 3.2 Microscopic oil displacement mechanism analysis

We conducted oil displacement experiments using various displacing systems after saturating the microfluidic chip with oil. Digital processing of the microscopic images enabled the segmentation of oil and water phases, allowing us to analyze residual oil distribution patterns and flow characteristics.

**FIGURE 9:**
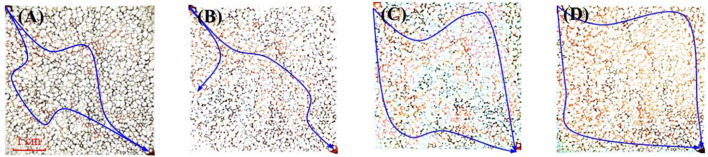
Microscopic images at breakthrough for different flooding systems: **(A)** water flooding, **(B)** SiO_2_ flooding, **(C)** HAWP flooding, and **(D)** HAWP + SiO_2_ flooding.

Figure 9 shows the sweep areas of different displacing fluids, where the red color denotes the displacing phase and black indicates the oil phase. Water flooding, SiO_2_ flooding, HAWP flooding, and HAWP + SiO_2_ flooding achieved sweep coefficients of 12%, 41%, 63%, and 91%, respectively. The results showed that water flooding exhibited channeling and failed to effectively sweep residual oil, while SiO_2_ flooding showed some improvement over water flooding. HAWP flooding achieved a higher sweep coefficient than water flooding due to its higher viscosity, which improved the water-oil mobility ratio. When HAWP was combined with SiO_2_, the viscosity increased, and SiO_2_ enhanced the displacement efficiency of HAWP.

**FIGURE 10:**
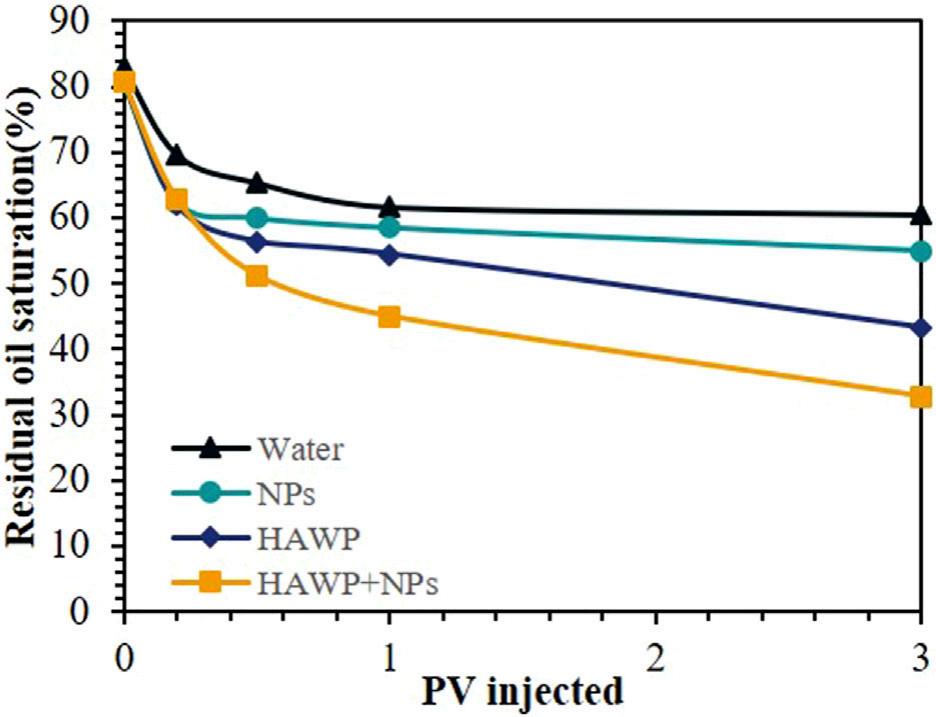
Relationship between residual oil saturation and injection volume for different flooding solutions.

Figure 10 shows the relationship between residual oil saturation and injection volume for different oil displacement systems. During the early stage of displacement (before 0.3 PV), water flooding showed a slow change in residual oil saturation, while the other three displacement methods showed similar residual oil saturation levels. As injection volume increased, the residual oil saturation ranked from highest to lowest in the following order: water flooding, SiO_2_ flooding, HAWP flooding, and HAWP + SiO_2_ flooding. In the later stages of displacement, the residual oil saturation curves for water flooding and SiO_2_ flooding became relatively stable, while HAWP flooding and HAWP + SiO_2_ flooding showed a steady declining trend.

**FIGURE 11:**
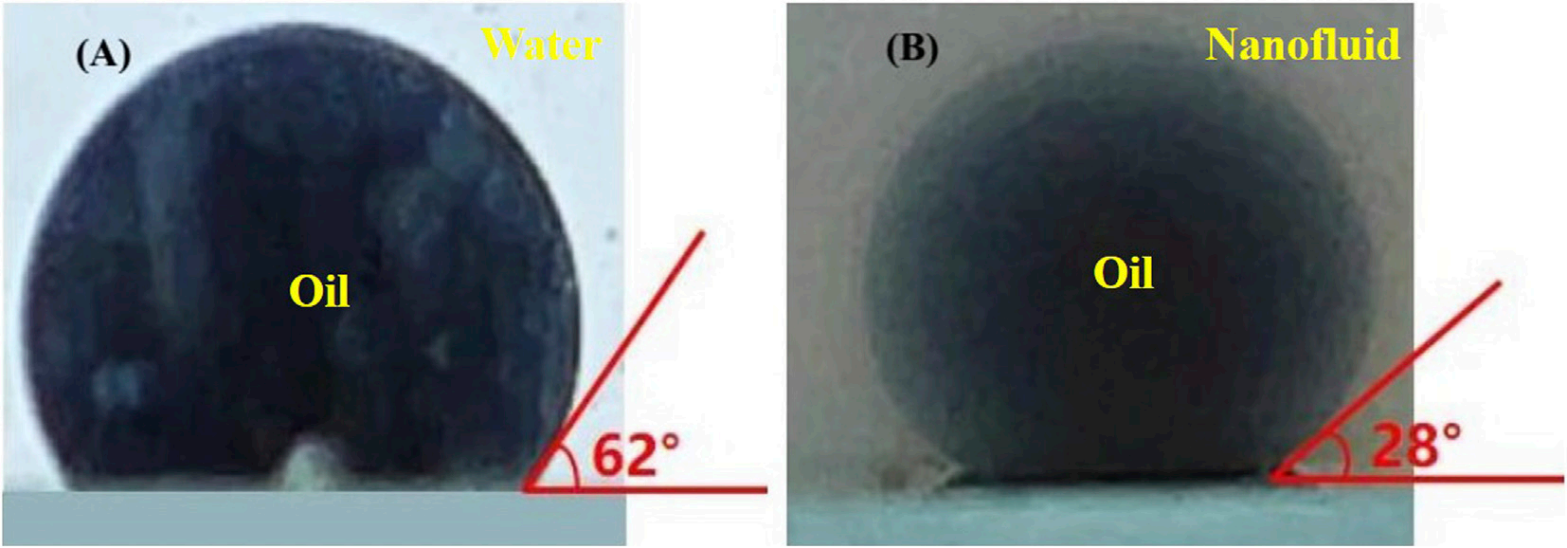
Oil-water contact angles of different systems: **(A)** formation water, **(B)** 2000 ppm HAWP +0.1wt% SiO_2_ composite system.

The enhanced performance of HAWP and HAWP + SiO_2_ flooding resulted from two factors. First, their higher viscosity led to increased sweep coefficients. Second, nano-SiO_2_ altered the wettability, as shown in Figure 11. The wetting angle of the oil phase has changed from 62° to 28°, which has improved the utilization degree of the residual oil and the displacement efficiency. improving residual oil mobilization and displacement efficiency. Moreover, the dragging effect of nanoparticles on crude oil and the reduction of interfacial tension also play a significant role.

Understanding residual oil flow characteristics helps elucidate flow mechanisms, quantify the production contribution from different residual oil types, and enhance recovery efficiency.

Based on the shape factor (*G*, defined as the area of a single residual oil block divided by the square of its perimeter), residual oil was classified into punctiform-like(0.56 < *G* < 1), membrane-like(0.34 < *G* < 0.56), cluster-like(0.06 < *G* < 0.34), and reticulated-like patterns(0 < *G* < 0.06).

**FIGURE 12:**
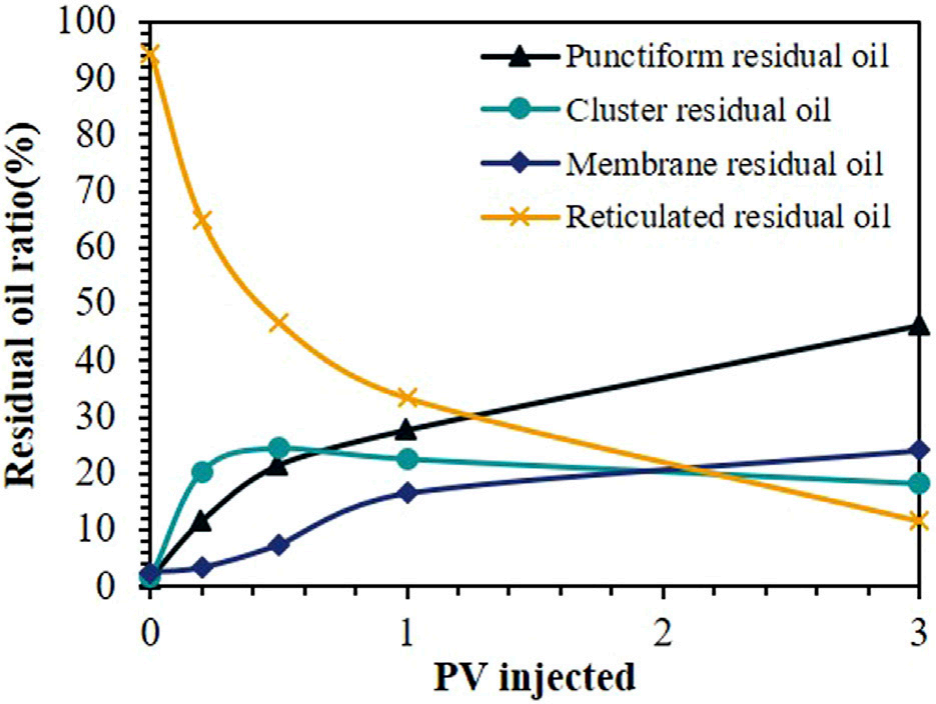
Proportions of different types of residual oil during the HAWP flooding process.

Figure 12 shows the variation trends in the proportions of different types of residual oil during the HAWP flooding process with increasing injection volume. The reticulated-like residual oil proportion demonstrates a consistent decline, whereas the cluster-like residual oil proportion shows a temporary increase followed by a decrease. Both membrane-like and punctiform-like residual oil proportions increase steadily. These trends reveal the transformation of residual oil during displacement: from reticulated-like to cluster-like patterns, and ultimately to membrane-like and punctiform-like configurations.

**FIGURE 13:**
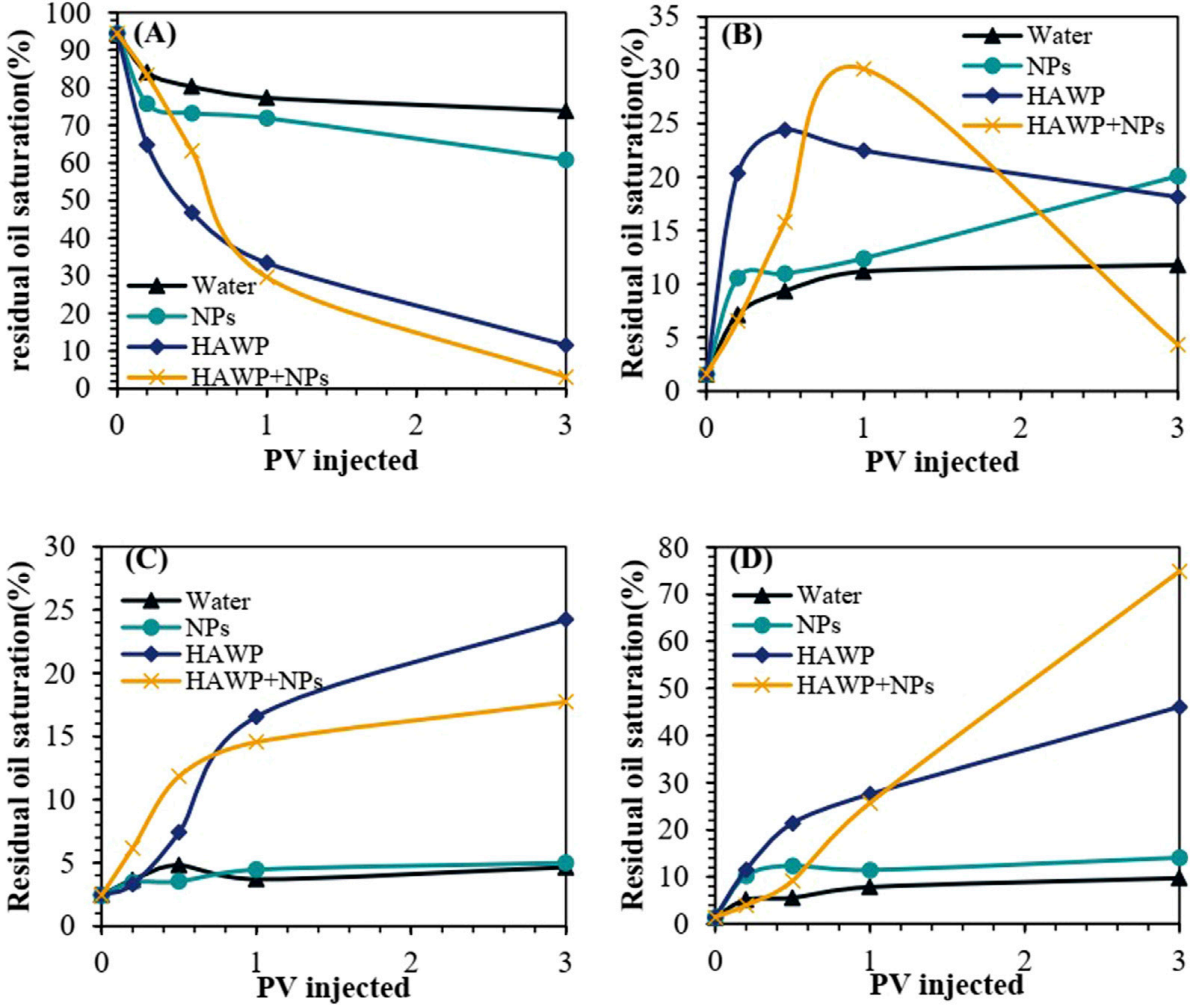
Changes in different types of residual oil content: **(A)** reticulated-like residual oil, **(B)** cluster-like residual oil, **(C)** membrane-like residual oil, and **(D)** punctiform-like residual oil.

Figure 13 shows the evolution of different residual oil types under various displacement systems. During the initial displacement stage, where web-like residual oil transforms into cluster-like residual oil, the transformation rates followed by SiO_2_, water, and HAWP + SiO_2_ in descending. As the displacement progresses, the proportions of different residual oil types remain relatively stable in water flooding and SiO_2_ flooding systems, predominantly consisting of reticulated-like residual oil. However, in HAWP and HAWP + SiO_2_ flooding systems, the residual oil continues to transform into membrane-like and punctiform-like patterns.

After injecting 3.0 PV of HAWP solution, reticulated-like residual oil accounts for 11.4%, while the proportions of cluster-like, membrane-like, and punctiform-like residual oil were approximately equal. The HAWP + SiO_2_ composite system achieves over 70% punctiform-like residual oil at the same injection volume. This indicated that the HAWP + SiO_2_ composite system shows a better transformation effect on residual oil, which is attributed to the nanoparticles' ability to adsorb onto the wall surface, improving the wall wettability and increasing the contact angle.

### 3.3 Analysis of oil displacement performance

**FIGURE 14:**
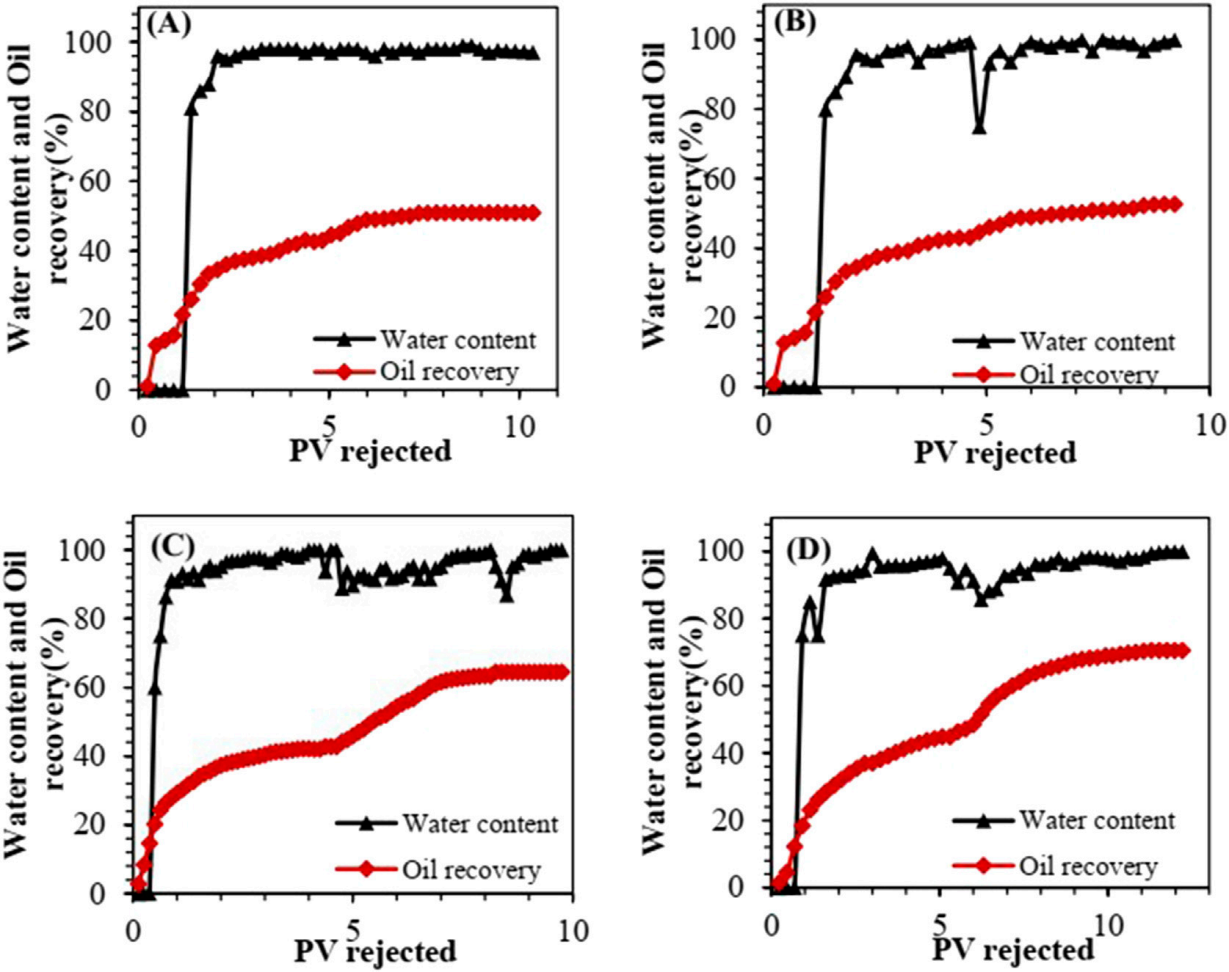
Recovery efficiency and water content profiles for different flooding systems: **(A)** continuous water flooding, **(B)** 0.1% SiO_2_ flooding, **(C)** 2000 ppm HAWP flooding, and **(D)** 2000 ppm HAWP +0.1% SiO_2_ flooding.

Figure 14 shows the variations in oil recovery and water content during the displacement process of different flooding systems.

For the continuous water flooding experiment, the water content remained near zero during the first 1.0 PV injection, while the oil recovery curve exhibited a significant increase. After 1.0 PV of formation water injection, water breakthrough occurred at the core outlet, resulting in the formation of high-permeability channels. Subsequently, the displacement efficiency decreased, leading to slow growth in oil recovery. As the formation water injection continued, the water content in the produced fluid increased rapidly, reaching 98% at 4.0 PV injection.

In the SiO_2_ flooding experiment, water flooding continued until the water content reached 98%. After switching to a 0.1 wt% SiO_2_ flooding system, the water content decreased initially but rose back to 98% after injecting 2.0 PV. The recovery curve showed a slight increase in slope after injecting 1.0 PV of this displacement system. This indicated that nanoparticle flooding showed moderate improvement over water flooding, though the effect was not significant. The improvement resulted from the nanoparticles' ability to reduce interfacial tension, alter wettability, and decrease crude oil viscosity.

In the HAWP flooding experiment, during the initial water flooding stage, the oil recovery increased rapidly while the water content remained relatively low, showing effective oil production performance. After injecting 4.0 PV of formation water, when the oil recovery was unchanged, the flooding system was switched to a 2,000 ppm HAWP solution. Similar to the initial water flooding stage, the early phase of HAWP flooding demonstrated a rapid increase in recovery efficiency accompanied by a significant decrease in water content. During the first 2.5 PV of HAWP injection, both the oil recovery and water content showed favorable performance. This enhancement can be attributed to the increased viscosity of the displacement fluid, which improved the mobility ratio and enhanced the sweep coefficient. We found that the water content would decrease after injecting 8 pore volumes (8 PV). This is mainly because the subsequent water flooding was adopted when injecting 8 pore volumes (8 PV). Before that, the displacement was carried out with 2,000 ppm HAWP. Due to its relatively high viscosity, its injection performance was not good. After switching to the subsequent water flooding, it was easier to inject, and there was still high-viscosity polymer remaining in the core. Subsequently, however, the water content began to increase again, leading to a gradual slowdown in recovery improvement.

In the combined HAWP + SiO_2_ flooding experiment, after 4.0 PV of water flooding, the water content of the produced fluid reached 98%. Upon switching to the 2,000 ppm HAWP +0.1wt% SiO_2_ displacement system, a rapid decrease in water content was observed, accompanied by an increased slope in the recovery efficiency curve, which was maintained for a considerable period. These results demonstrate that this composite system effectively displaced the residual oil in the core. The enhanced oil recovery performance can be attributed to two main mechanisms: firstly, the increased viscosity of the system improved the water-oil mobility ratio, thereby enhancing the macroscopic sweep coefficient; secondly, the system’s favorable surface activity and low interfacial tension facilitated the detachment of oil droplets from the rock surface, resulting in improved microscopic displacement efficiency.

**FIGURE 15:**
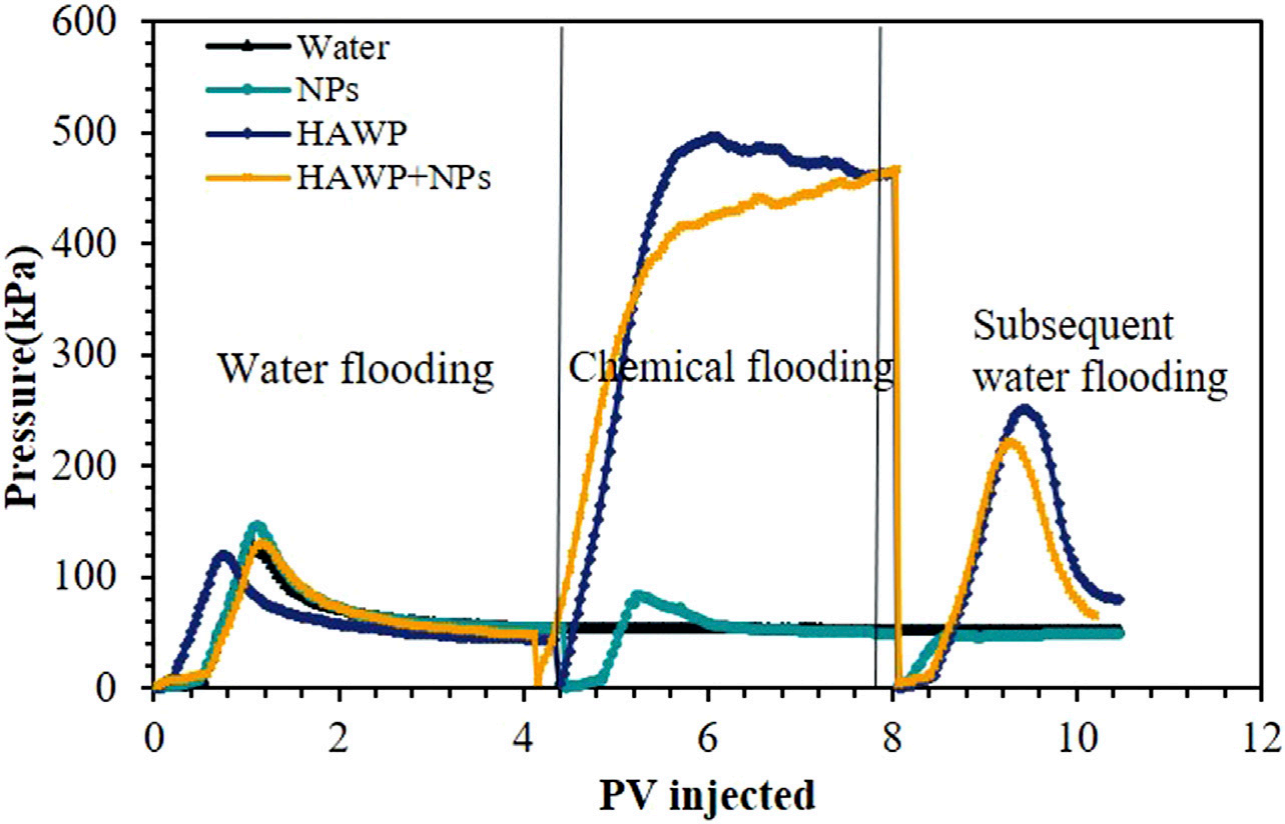
Injection pressure variation curves of different flooding systems.

The results indicate that a significant amount of crude oil was displaced during the initial water flooding stage. As water flooding continued, the increment in recovery efficiency gradually decreased. After switching to chemical flooding, both 2,000 ppm HAWP and 2,000 ppm HAWP +0.1wt% SiO_2_ systems demonstrated notable enhancement in oil recovery. The composite system achieved an additional 6% increase in recovery efficiency compared to the polymer flooding system. As shown in Figure 15, during the displacement experiments, we found that the 2000 ppm HAWP +0.1 wt% SiO_2_ systems could reduce the injection pressure by up to 14% compared with traditional polymers.

## 4 Conclusion

(1) We compounded an optimal polymer/nanoparticle composite flooding system through rheological testing. The system consists of 2,000 ppm hydrophobically associating water-soluble polymer (HAWP) solution and hydrophilic SiO_2_-nanoparticles at a mass concentration of 0.1wt%.
(2) Basic performance tests demonstrate that the addition of SiO_2_ nanoparticles increases the polymer solution viscosity by over 76%. Interfacial tension measurements show that the nanoparticles effectively reduce interfacial tension. SEM image confirms that nanoparticles adsorb onto polymer molecular chains to form network structures, which explains the microscopic mechanism behind the improved viscosity of the composite system.
(3) Microscopic visualization displacement experiments reveal the oil displacement mechanism of the polymer/nano-SiO_2_ composite system. Compared to water flooding and polymer flooding, the polymer/nano-SiO_2_ composite flooding improves the sweep coefficient by 79% and 28%, respectively. It promotes the evolution of residual oil phase structure from reticular to cluster, film, and spotted patterns, enhancing displacement efficiency and achieving effective oil recovery.
(4) Core flooding experiments confirm the nanoparticle composite flooding system’s enhanced oil recovery and pressure reduction effects. Under the same injection volume, polymer/nano-SiO_2_ composite flooding achieves higher recovery efficiency than both water flooding and polymer flooding. As the displacement process continues, the recovery efficiency shows an increasing trend, achieving a 6% higher recovery than polymer flooding while reducing injection pressure by up to 14%.

## Data Availability

The original contributions presented in the study are included in the article/supplementary material, further inquiries can be directed to the corresponding author.
